# Editorial: Advances in diabetes and hypertension research

**DOI:** 10.3389/fendo.2025.1711423

**Published:** 2025-10-13

**Authors:** Christiano Argano, Rosario Scaglione

**Affiliations:** ^1^ Department of Internal Medicine, National Relevance and High Specialization Hospital Trust ARNAS Civico, Di Cristina, Benfratelli, Palermo, Italy; ^2^ Department of Health Promotion Sciences, Maternal and Infant Care, Internal Medicine and Medical Specialties, [PROMISE], University of Palermo, Palermo, Italy

**Keywords:** diabetes mellitus, hypertension, biomarkers, novel lipid indicators, proteomics, cardiovascular risk, meta-analysis, exercise

Diabetes and hypertension place a significant burden on health care systems (1). In 2024, 589 million adults (aged 20–79), equaling 1 in 9 subjects, were living with diabetes worldwide. This number is predicted to rise to 853 million by 2050 (2). Diabetes and its comorbidities often lead to increased use of primary care and hospital services (3), representing a major cause of death and resulting in an estimated $1.015 trillion in global health expenditures in 2024 (2).

On the other hand, the number of individuals with hypertension doubled between 1990 and 2019, rising from 650 million to 1.3 billion (4). Hypertension can lead to stroke, heart attack, heart failure, kidney damage, and other diseases (5). It is worth noting that only 21% of individuals with hypertension have it under control, and achieving cardiovascular protection remains to date one of the most important goals of antihypertensive treatment (6). 

It is well known that diabetes and hypertension are closely linked conditions that share common risk factors such as obesity, insulin resistance, and inflammation, often occurring together (7). Hyperglycemia can damage blood vessels and kidneys, which elevates blood pressure, while hypertension itself can increase the risk of developing diabetes (7). These comorbidities significantly increase the risk of cardiovascular, cerebrovascular and kidney diseases, requiring integrated management that includes lifestyle changes and medications and a holistic approach to patient care (8).

Consistent with this goal, this Research Topic aimed to shed light on various aspects of the relationship between diabetes and hypertension presenting new insights into the potential pathological mechanisms underlying these relationships.

Overall, 3 meta-analyses, 2 prospective studies, 8 studies focused on different aspects of diabetes and 8 works focusing on the diabetes-hypertension association have been published.

Wang et al. analyzed proteomic data using two-dimensional gel electrophoresis and electrospray-quadrupole time-of-flight MS/MS in 80 obese patients, 76 patients with newly diagnosed type 2 diabetes mellitus (T2DM) combined with obesity, and 73 healthy controls, in light of several studies indicating the need to identify increasingly reliable serum markers for diagnosing and tracking the progression of diabetes. Several proteins were found to be differentially expressed, with α2-macroglobulin (α2-MG) showing significant upregulation in the obesity and T2DM+obesity groups and higher α2-MG levels in the obesity and T2DM+obesity groups compared to the control group. These data suggest that α2-MG levels are highly sensitive and specific for predicting obesity and T2DM, indicating their potential as a T2DM diagnostic biomarker.

Peng et al. evaluated the association between ALDH2 and diabetes risk by analyzing data from 4,535 participants in the China Cardiometabolic Disease and Cancer Cohort Study. The authors considered studies that have linked genetic variations, such as the ALDH2 rs671 genotype variant, to an increased risk of developing diabetes in the Asian population (9). Among male participants, the ALDH2 rs671 GA/AA genotype was associated with a lower diabetes risk than the GG genotype, even after adjusting for alcohol consumption and other potential confounders. Subgroup analyses revealed that this protective effect was most pronounced in individuals with a BMI < 24. Abdominal adiposity accounted for 30.4% of the ALDH2–diabetes association, and BMI mediated 18.9% of this relationship; however, alcohol consumption showed no significant mediating effect (p = 0.56). These findings revealed that East Asian men with the ALDH2 GG genotype were at an increased risk for diabetes compared to those with the GA/AA genotype, particularly those with a BMI < 24. Thus, individuals with the GG genotype, even with a normal BMI, may benefit from exercise and dietary interventions able to reduce waist circumference.

In light of data implicating adropin, a newly identified peptide hormone expressed in the liver, brain and kidney, in processes such as lipid metabolism and inflammation, which are involved in the development of diabetic nephropathy (10, 11), Chen et al. measured serum adropin levels in eighteen patients with early chronic kidney disease (CKD) and forty patients with advanced CKD. Nine subjects without diabetes were studied as a control group. Subjects with T2DM had significantly higher adropin levels than controls. T2DM patients with advanced CKD had higher adropin levels than those with early CKD. Among T2DM patients, subjects who experienced CKD progression had higher adropin levels than those without. Thus, adropin predicts CKD progression in T2DM patients with 86% sensitivity and 70% specificity at a cutoff value of 6872.24 pg/ml. The association with CKD progression was still significant after adjusting for age, gender and body mass index.

These findings suggest that serum adropin could serve as a potential biomarker for predicting CKD progression in subjects with T2D.

Feng et al. examined the association between sleep-disordered breathing (SDB) and the risk of metabolic syndrome (MetS) in various gender, age, and symptom subtype groups. The authors identified the nocturnal hypoxia parameter that best reflects this relationship and assessed the connection between sleep variables and MetS. Combining these parameters into a sleep quality score could improve predictions of health outcomes. Participants were monitored using the Type IV sleep monitoring device and completed structured questionnaires.

The severity of SDB was independently associated with an increased risk of MetS, particularly in men under 60 and women aged 60 and above. A total of 1,483 SDB patients were grouped into four distinct clusters: Cluster 1 included the pure insomnia group with fewer daytime symptoms; Cluster 2 consisted of the minimally symptomatic group; Cluster 3 comprised the insomnia group with multiple daytime symptoms; and Cluster 4 encompassed the group with upper airway symptoms and sleepiness. Among the SDB subtypes, there was no significant difference in the prevalence of metabolic syndrome. However, the pure insomnia group had the highest prevalence of hypertension.

These findings suggest the relevant role of gender, age differences, and sleep symptom subtypes when evaluating SDB and MetS. This approach would indicate that early identification and consideration of different subtypes are necessary to optimize treatment for these subjects.

Yan et al. conducted a network meta-analysis to evaluate the efficacy of aerobic training (AT), resistance training (RT), combined training (AT+RT), high-intensity interval training (HIIT), and traditional Chinese exercises (TCEs) on glycemic control, lipid profiles, and weight management in prediabetic subjects. This marks the first time HIIT and TCEs have been included in such an assessment. A total of 74 studies involving 5,683 participants were included. The results showed that HIIT was the most effective in reducing hemoglobin A1c [Surface Under the Cumulative Ranking (SUCRA) 84.3%], and increasing high-density lipoprotein (SUCRA 87.3%). AT+RT was the most effective in reducing total cholesterol (SUCRA 98.3%), TG (SUCRA 99.9%), low-density lipoprotein (SUCRA 82.2%), and body mass index (SUCRA 66.4%). TCEs showed the most significant improvements in reducing 2hPG (SUCRA 83.5%), body weight (SUCRA 79.1%), and waist circumference (SUCRA 84.6%). These results indicate that various exercise interventions can significantly improve glycemic and lipid profiles in prediabetic patients and that HIIT and AT+RT were found to be the most effective interventions. These findings provide the latest evidence supporting exercise interventions for managing prediabetes.

Yang et al. studied the effects of SY-009, a novel SGLT1 inhibitor, on plasma metabolomics in patients with T2DM and the potential metabolic regulatory mechanisms involved. A total of 50 participants with T2DM were enrolled and randomly assigned to one of five dose groups: > 0.5 mg BID, 1 mg BID, 2 mg BID, 1 mg QD, and 2 mg QD. Within each group, participants were randomly assigned a 4:1 ratio to receive either the SY-009 capsule or a placebo. SY-009 caused a series of postprandial plasma metabolite changes in patients with T2DM, especially significant changes in the bile acid profile. These changes provide a new perspective on how SY-009 lowers blood glucose.

Xue et al. performed a meta-analysis to obtain a comprehensive overview of the differences between once-weekly basal insulin (including icodec and basal insulin Fc) and once-daily basal insulin (including glargine and degludec) in patients with T1DM and T2DM.

Currently, novel basal insulin analogs that can be administered once weekly by subcutaneous injection have been developed to improve treatment acceptance and adherence. Icodec and basal insulin Fc (12–14) are the two most advanced once-weekly basal insulins for the treatment of patients with T1DM and T2DM. The authors evaluated a total of 12 studies, comprising 5,895 patients, of whom 3,104 (52.7%) used once-weekly insulin and 2,791 (47.3%) used once-daily insulin. In the pooled data, glycated hemoglobin changes from baseline to the end of the trial demonstrated significantly good glycemic control in the once-weekly insulin group, especially in insulin-naïve T2DM patients or those using icodec. Once-weekly insulin was correlated with a higher risk of level 1 hypoglycemia. There was no significant difference in fasting plasma glucose, time in range, or level 2 or 3 hypoglycemic events.

These data indicate that once-weekly basal insulin is safe and effective in a slight decrease of HbA1c with similar levels of grade 2 and 3 hypoglycemic events compared to once-daily insulin, although the risk of level 1 hypoglycemia and weight gain increased.

Chen et al. retrospectively analyzed the data on clinical presentations, laboratory results, and cranial CT and MRI scans of six patients with diabetic striatopathy (DS), a rare disorder with clinical manifestations of hemichorea, non-ketotic hyperglycemia, and high signal intensity on MRI scans or high density on CT scans in the basal ganglia. These manifestations are typically associated with poor glycemic control. The goal of the study was to raise awareness among physicians about this rare neurological manifestation that is often overlooked in patients with diabetes.

All six patients investigated complained of involuntary unilateral movements, which primarily affected the arm and leg. Case 3 showed bilateral caudate nucleus hyperdensities on the CT examination, while the other 5 patients showed unilateral caudate nucleus hyperdensities. In addition, five patients (except Case 5) underwent MRI, all showing hypersignal lesions on T1-weighted images. Case 6 exhibited a low signal in the right basal ganglia on MRI susceptibility-weighted imaging sequences. All six patients exhibited carotid artery or cerebral artery stenosis. Following strict blood glucose control and symptomatic management, the symptoms of chorea improved significantly in all patients, and repeat imaging studies indicated that the lesions gradually disappeared. In conclusion, DS may occur in patients with ketotic hyperglycemia. Typical movement disorders do not always coincide with typical imaging results. The authors speculated that poor vascular conditions and marked hyperglycemia promote the development of DS. It is recommended to test blood glucose levels at the time of the diagnosis of hemichorea.

Zhou et al. analyzed the mediating role of the atherogenic index of plasma (AIP), a novel biomarker (15), in the relationship between multiple obesity indices (BMI, WHR, WHtR, and BAI) and the prevalence of diabetes in hypertensive subjects. This cross-sectional study from the China Multi-Ethnicity Cohort study suggested that obesity indices were significantly higher in diabetic patients compared to those without. Moreover, logistic regression analysis suggested that higher obesity index quartiles were associated with an increased risk of diabetes in both crude and adjusted models (p < 0.05). Mediation analysis revealed that the association between obesity and the risk of diabetes, mediated by BMI, WHR, waist-to-height ratio (WHtR), and body adiposity index (BAI), through the AIP was 17.2%, 15.3%, 15.8%, and 19.2%, respectively. These data are consistent with the indication that the AIP significantly mediates the association between each of the four obesity indices and diabetes prevalence in hypertensive patients.

Meng et al. investigated the potential correlation between the non-high-density lipoprotein cholesterol-to-high-density lipoprotein cholesterol ratio (NHHR) and the risk of diabetes and prediabetes in adults with hypertension. The NHHR is an emerging composite lipid marker that offers superior predictive value for cardiovascular disease, metabolic syndrome, fatty liver disease, and certain renal diseases.

In this cross-sectional survey, 10,250 hypertensive patients, including 2,198 with diabetes and 4,138 with prediabetes, were screened from National Health and Nutrition Examination Survey (NHANES)-collected data from 2009 to 2018.

The fully adjusted model indicated that each unit increase in NHHR was associated with a 21% higher risk of diabetes or prediabetes. In patients with hypertension, the NHHR was positively correlated with the prevalence of diabetes and prediabetes, with a nonlinear trend in the fitted curve (nonlinearity, P = 0.007). These data indicate that the NHHR is positively and non-linearly correlated with diabetes and prediabetes in patients with hypertension, particularly in women. This may support the use of NHHR as a valuable tool to identify patients at high risk for these conditions.

Jin et al. brought new insights into the role of novel lipid-related markers as predictors of T2DM risk. The lipid accumulation product (LAP) (16) may be considered a more accurate indicator of the metabolic risks associated with visceral obesity and excessive lipid accumulation. The Chinese Visceral Adiposity Index (CVAI) (17) has been reported as a surrogate biomarker for assessing visceral fat accumulation. In view of this, the authors analyzed the role of T2DM in the associations of WC, BMI, LAP, CVAI, and triglyceride-glucose (TyG) index.

A total of 1,965 hypertensive subjects aged 45 years and older were included in the cross-sectional analysis, and 1,576 hypertensive subjects without T2DM were included in the cohort analysis. The risk of T2DM increased significantly with higher quartiles of WC, BMI, LAP, CVAI, and TyG (all P-trends < 0.001).

In the cohort study, the Cox regression model showed that WC, BMI, LAP, CVAI and TyG were associated with a higher risk of incident T2DM. ROC analysis revealed that the TyG index had the strongest area under the curve. These data indicate that higher levels of WC, BMI, LAP, CVAI, and TyG are associated with a higher risk of developing incident T2DM in elderly Chinese hypertensive patients, and that TyG may be the most effective predictive indicator.

Ren et al. analyzed the association between the TyG index and hypertension based on a cross-sectional study from the ongoing REACTION study in China and a meta-analysis of epidemiological studies. A total of 4,177 participants aged 58.62 ± 8.40 years were included. The TyG index was significantly associated with a higher likelihood of hypertension, and the association was present in both isolated systolic hypertension and systolic-diastolic hypertension, but not in isolated diastolic hypertension. Moreover, data from a meta-analysis of 34 relevant studies were included. These revealed a positive association between the TyG index and hypertension. These data indicate that a higher TyG index is significantly associated with a higher risk of clinical hypertension, which may provide new insights into the clinical management of hypertension.

In their prospective study, Zhao et al. utilized data from the Bogalusa Heart Study (18) to investigate the relationship between changes in the TyG index during childhood and pre-hypertension in adulthood based on a positive correlation between the TyG index and blood pressure, indicating that a high TyG index is related to a higher risk of developing pre-hypertension and hypertension. Data on triglycerides, fasting glucose, and low-density lipoprotein cholesterol were collected from cross-sectional examinations of participants during childhood. Blood pressure in adulthood was subdivided into normotensive and pre-hypertensive groups. Logistic regression was employed to evaluate the relationship between the TyG index in childhood and pre-hypertension in adulthood.

A total of 1,222 participants were included in the study, of whom 258 presented with pre-hypertension in adulthood. A significant association was found between an increase in the TyG index in childhood and pre-hypertension in adulthood, when the data were stratified by ethnicity and gender. Each unit increase in the TyG index was associated with a 70% increased likelihood of pre-HTN among American Caucasian participants and a 90% increased likelihood among male participants, independent of potential confounders. This suggests that a high TyG index may be a robust predictor of pre-HTN events in these groups. Accordingly, monitoring of the TyG index may help in screening subjects at higher risk for pre-hypertension.

Lin et al. analyzed the association between the comorbidity of gestational diabetes mellitus (GDM) and hypertensive disorders of pregnancy (HDP) to explain adverse birth outcomes often presenting in women with these conditions. The data came from the electronic medical record system (EMRS) of the Zhoushan Maternal and Child Health Hospital. A total of 13645 pregnant women were included. GDM+HDP was significantly associated with a higher risk of composite adverse neonatal outcomes, including preterm birth, placenta previa, and/or neonatal jaundice, and a higher risk of small for gestational age and large for gestational age compared with the normal group. In addition, HDP diagnosed in the 21st-27th week, comorbid with GDM, was associated with the lowest gestational age at delivery (P = 0.0002) and birth weight (P = 0.0138). Moreover, combined hyperglycemia comorbid with HDP had the strongest association with reduced gestational age (β= -0.83, P = 0.0021).

These results suggest that pregnant women suffering from both GDM and HDP are at a higher risk for adverse neonatal outcomes. Accordingly, the prevention and treatment of GDM and HDP, especially their comorbidity, have to be prioritized in pregnant women.

Yang et al. reported that time systolic blood pressure in the target range (SBP-TTR) is a new metric for evaluating blood pressure control, which refers to the proportion of time that SBP remains within the target range (19). This index has been reported as an independent risk factor for stroke, and it would be particularly useful for monitoring all those conditions in which a controlled reduction in pressure is necessary.

The authors included 28,591 participants, both with and without diabetes [mean age, 57.5 years; 83.8% men; 23.2% with diabetes] from the Kailuan Study. After a median of 8.7 years of follow-up, 2,206 stroke cases occurred. Among subjects with diabetes, those with an SBP-TTR of 75%–100% had a lower risk of stroke compared to those with an SBP-TTR of 0%–25%. Among subjects without diabetes, those with an SBP-TTR of 50%–75% had a significantly lower risk of stroke. A significant interaction was found between diabetes status and SBP-TTR. These results are consistent with the indication that a higher SBP-TTR is associated with a reduced risk of stroke in subjects with or without diabetes. These findings emphasize the importance of maintaining blood pressure within the target range to mitigate stroke risk. This requires strong blood pressure management in diabetic patients.

Zheng et al. evaluated the role of human urinary kallidinogenase (HUK) (20, 21) in reducing the incidence of progressive ischemic stroke (PIS) in patients with acute ischemic stroke (AIS), particularly in a subgroup with vascular pathology and thrombolytic treatment. In total, 916 patients with AIS were included in the study. The patients were divided into two groups: one that received HUK treatment in addition to standard care and one that received standard care alone. In addition, subgroup analyses were conducted based on stroke subtype (TOAST classification), thrombolytic treatment, and infarction location.

HUK treatment significantly reduced the incidence of PIS (p < 0.001), with the most notable effects observed in patients with large-artery atherosclerosis and small-artery occlusion, those not undergoing intravenous thrombolysis, and those with anterior circulation infarctions. Conversely, no significant reduction was noted in patients with cardioembolic stroke.

These promising data indicate that HUK treatment is an effective strategy for reducing the risk of PIS in patients with AIS, particularly those at higher risk owing to specific vascular pathologies. These findings support the use of HUK in clinical practice to improve the outcomes of patients with stroke.

Taken together, the studies published in this Research Topic increase the reader’s understanding of the interactions between diabetes and hypertension, update their knowledge, particularly concerning new pathophysiological aspects, and identify novel biomarkers that can be used to explore the increased cardiometabolic and cerebrovascular risk in clinical practice.
